# Exploring multiple electrical layers overlying coal seams using the transient electromagnetic method

**DOI:** 10.1371/journal.pone.0273423

**Published:** 2022-10-24

**Authors:** Yang Yang, Siqin Liu, Fangbo Chen, Hanbo Chen, Tianyu Zhang, Yu Han, Mingkun Jia

**Affiliations:** 1 College of Earth Sciences, Guilin University of Technology, Guilin, Guangxi, China; 2 School of Geosciences and Info-Physics, Central South University, Changsha, Hunan, China; 3 School of Geophysics and Information Technology, China University of Geosciences, Beijing, China; 4 Geophysical Survey Center, China Geological Survey, Langfang, Hebei, China; 5 Hebei Institute of Geological Surveying and Mapping, Langfang, Hehei, China; Massachusetts Institute of Technology, UNITED STATES

## Abstract

The transient electromagnetic method (TEM) is widely applied in coal hydrogeological exploration owing to its sensitivity to low-resistivity bodies. However, when a coal seam is buried deep, particularly if there are multiple electrical layers in the vertical direction of the overlying stratum, the results of the calculation using the late-channel empirical formula of the TEM may no longer reflect the actual situation. In this study, we evaluated the principle of the one-dimensional (1D) Occam algorithm and the steps for performing an inversion. We proposed various two-, three-, and four-layer electrical models for inversion using the 1D Occam algorithm. Our results are consistent with the electrical distribution of the models, thus indicating the effectiveness of the algorithm. A test project of large-depth transient electromagnetic exploration in the Datong Coalfield in Shanxi, China, was selected for experimental verification. The 1D Occam inversion was used to successfully identify various electrical strata overlying the coal seam.

## 1. Introduction

The transient electromagnetic method (TEM) is an electromagnetic induction method using which the geoelectric information of an underground geological body can be obtained [1–4]. However, when the target strata are buried deep and there are multiple geological layers with different electrical properties, the TEM late-channel apparent resistivity calculated using the corresponding formula cannot be used to extract the geoelectric information from the data. With the development of sophisticated computational algorithms, it has become possible to extract more realistic geoelectric information using inversion programs [5]. Therefore, it is necessary to apply inversion technology to obtain meaningful information by detecting multiple electrical layers.

The application of the TEM one-dimensional (1D) inversion began in the 1980s. Based on the damped least squares method, Raiche et al. [6] carried out a joint inversion of the overlapping loops of the TEM and data collected by the Schlumberger device using the resistivity method. Huang and Wang used the damped least squares method to achieve 1D inversion of airborne electromagnetic data in the time domain. Both the algorithms apply layered medium parameterized inversion via which the layer thickness and resistivity can be inverted simultaneously. The inversion results depend on the number of layers and initial model [7]. Farquharson and Oldenburg added the characterization term of the degree of deviation between the model to be determined and a reference model in the objective function, thereby setting the number of inversion model layers to be higher than the number of observation data and the thickness of each layer of the medium to be fixed, after which the resistivity of each electrical layer can be inverted [8]. Auken introduced lateral constraints in 1D inversion and successfully solved the problems of poor lateral continuity of resistivity and layer thickness in the inversion profiles and improved the resolution of thin layers [9]. Sudha et al. used an inversion program based on the damped least squares method and Occam algorithm to carry out a joint inversion of the loop-source TEM and long-offset TEM, which solved the problem of the interface of inversion depth layer not being smooth [10].

These scholars have achieved good results. In addition, some scholars have performed TEM inversion with multiple electrical layers overlying the coal seams. For example, Khan et al. used short-offset transient electromagnetic technology and 1D Occam inversion to investigate the groundwater in-rush zone, which provides key information for the interpretation of the depositional facies of coal-bearing sequences [11]. The Occam algorithm, also known as the smoothest model inversion method, was proposed by Constable et al. [12]. It uses the initial model to fit the decay curve of the surveying points in the forward process and continuously adjusts the model to ensure that the fitting difference meets the accuracy requirements with low roughness and fast convergence speed. Furthermore, the algorithm reasonably selects the regularization parameter and iterative step size [13,14], which can clearly reflect the high- and low-resistivity layers, whereas the inverse resistivity profile is clearly layered, and the layer thickness can be precisely controlled.

In this study, we first utilized the synthetic models with 2–4 layers and validated the results with inverted TEM data using 1D Occam inversion to detect multiple electrical layers. Thereafter, using an engineering project in Datong Coalfield, Shanxi Province, China, we found that the use of 1D Occam algorithm to invert TEM data in the exploration of multiple electrical layers overlying coal seams produced an ideal effect.

## 2. Methodology

### 2.1. Calculation of apparent resistivity in loop-source TEM

**Fig 1:**
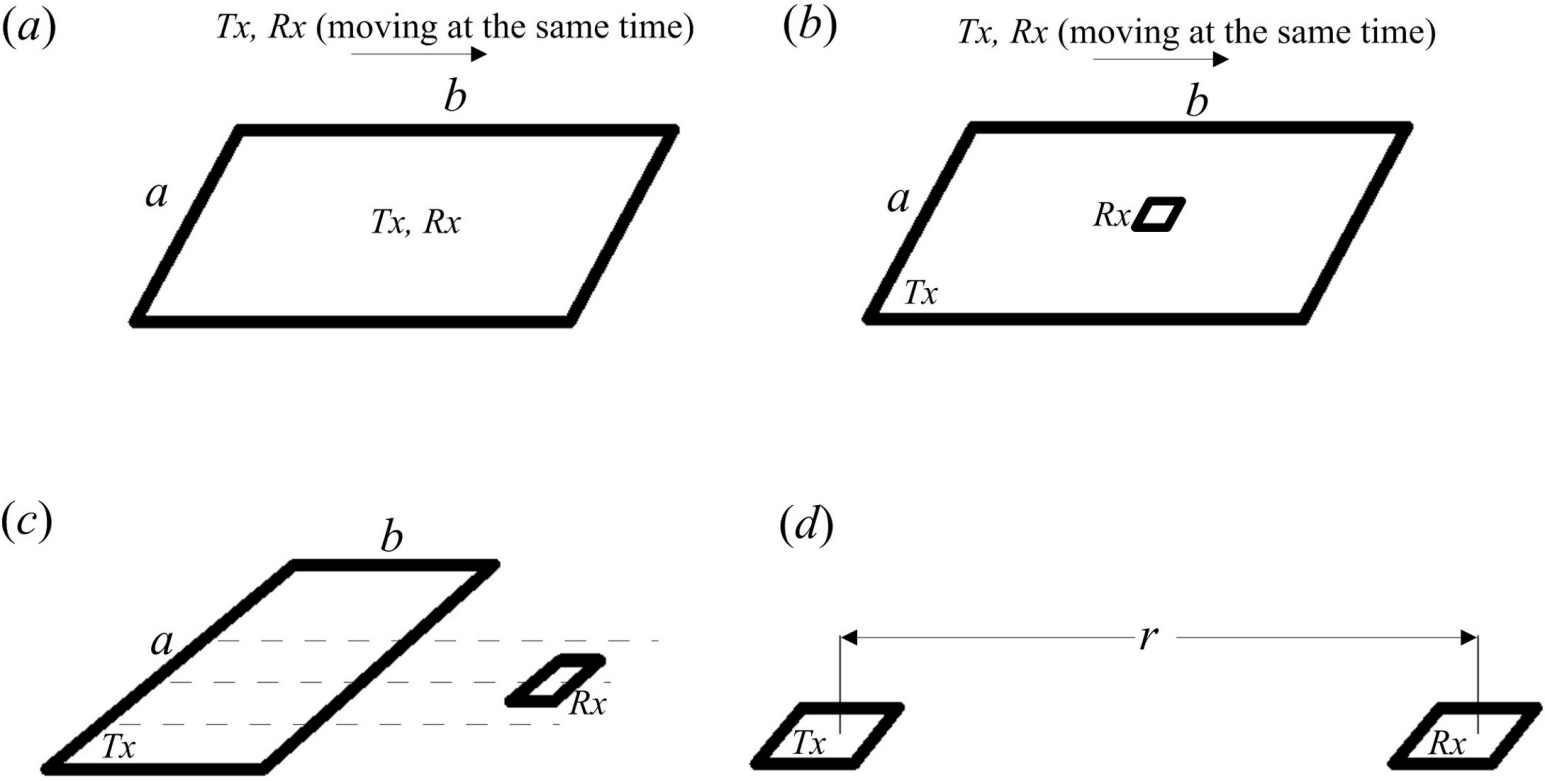
Observation devices of the transient electromagnetic method utilizing a magnetic couple source. (*a*) Overlapping loop device; (*b*) center loop device; (*c*) large fixed-source loop; (*d*) dipole device.

The TEM is a type of time-domain electromagnetic method. The transmitting device can be either a galvanic couple source (an electric dipole source with both ends grounded) or a magnetic couple source (an ungrounded loop source). When using a galvanic couple source, the long-offset [5,15] and short-offset TEM methods [16,17] can be implemented. When using a magnetic couple source, the device can be divided into overlapping loops, center loops, large fixed-source loops, and dipole devices (Fig 1).

The center loop device, which requires the receiving device to be located in the center of the transmitting loop, is most commonly used in TEM explorations. When the side length of the transmitting loop is large, the working efficiency of the center loop device is significantly reduced (Fig 1B). Further, a large fixed-source loop device can be observed inside and outside the loop (Fig 1C), but the resistivity obtained is unstable because of the sign change phenomenon outside the transmitting loop.

**Fig 2:**
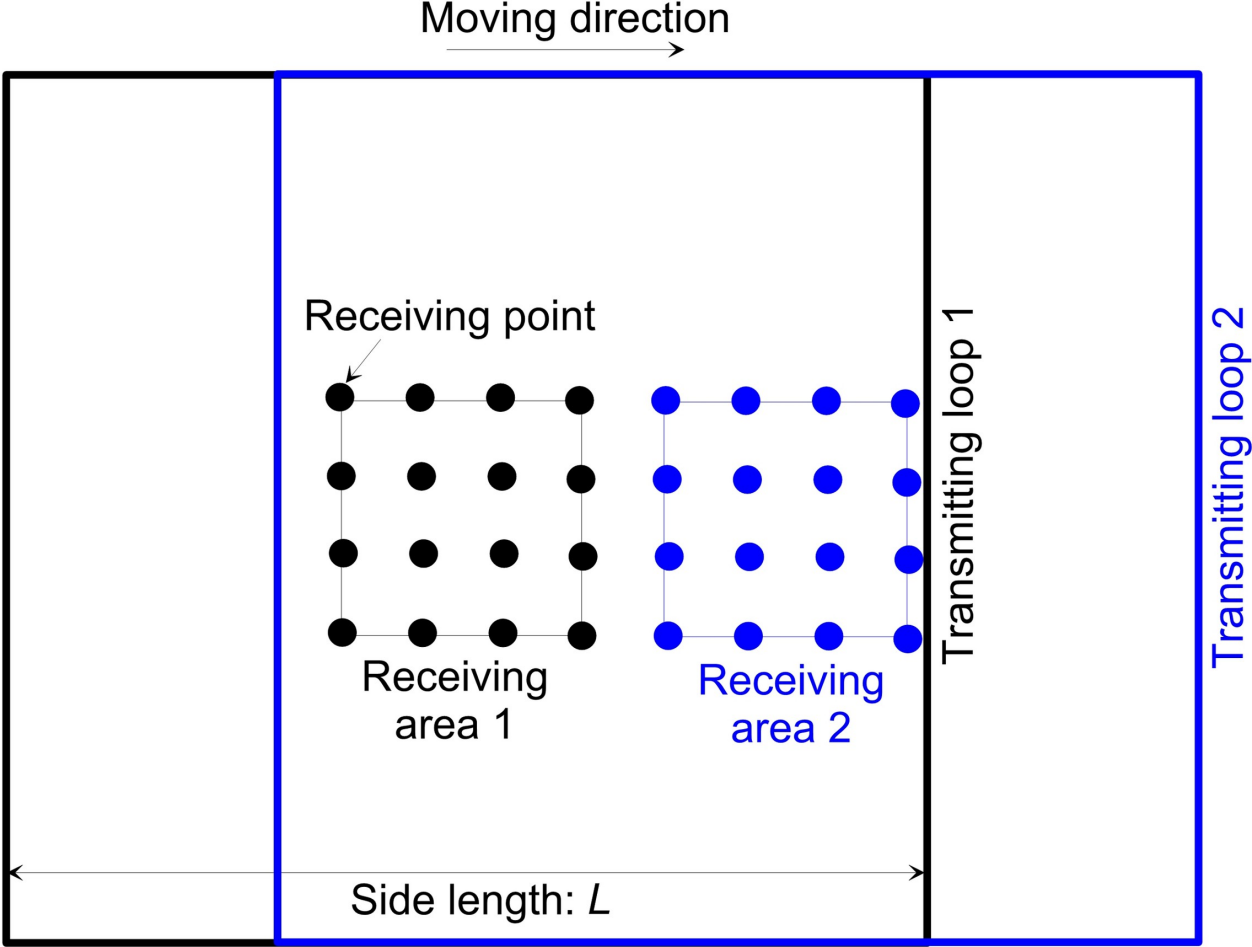
Schematic diagram of the working device.

A method was proposed to improve the detection technology of the loop-source TEM, which was to improve the working efficiency and interpretation accuracy of the loop-source TEM, in accordance with the application scope and characteristics of the two devices of the center and large fixed-source loops [18]. In this proposed method, the large fixed-source loop with a side length of 400–1,000 m was used in the transmitting part; the observation was carried out within a certain range of the central area inside the transmitting loop, and the portable receiving probe or coil was used to carry out observations along the surveying lines. This is called the “improved center loop method.” We evaluated this method, and the observation was conducted within one-ninth of the area inside the loop (i.e., the side length of the observation area was one-third of that of the transmitting loop; Fig 2).

In TEM exploration, the formula for apparent resistivity *ρ* can be expressed as follows [19–21]:

$$\rho =\frac{\mu_{0}}{4\pi t}{\left(\frac{2\mu_{0}S_{\text{T}}S_{\text{R}}}{5t(V(t)/I)}\right)}^{2/3},$$ (1)

where *μ*_0_ is the vacuum permeability and its value is (4π × 10^−7^) H/m; *S*_T_ is the area of the transmitting loop; *S*_R_ is the area of the receiving coil; *t* is the sampling time; *V*(*t*)/*I* is the normalized induced electromotive force.

Eq (1) presents the empirical formula for the late-channel apparent resistivity, and the corresponding apparent depth *h* can be calculated as follows:

$$h=\sqrt{\frac{\rho (t_{i})\times t_{i}}{2\mu_{0}}}$$ (2)

On this basis, with the apparent resistivity *ρ* and apparent depth *h* as parameters, the apparent resistivity–apparent depth profile along the survey line can be drawn. It is worth noting that only the distribution of “apparent resistivity” can be obtained using the two formulas, but the real geoelectric characteristics cannot be determined when exploring targets with large depths and multiple electrical layers.

### 2.2. Occam inversion method

#### 2.2.1. Basic principles

The basic concept of the Occam algorithm is to obtain the smoothest model that can fit the data by introducing a regularization term for model roughness into the objective function [22,23]. The objective function of the inversion is set as follows:

$$U=R+\mu^{-1}[{\left\|W(d-F(m)\right\|}^{2}-\chi_{*}^{2}],$$ (3)

where the first term is the fitting difference function between the measured data and forward model response, *μ* is the regularization factor, ***W*** is the data covariance weighting function, ***d*** is the data vector, ***m*** is the inversion model parameter, *F*(***m***) is the TEM forward model response corresponding to the model, $\chi_{*}^{2}$ is the artificially set target fitting difference, and *R* is the longitudinal roughness function of the inversion model, which can be expressed as:

$$R={\left\|\partial m\right\|}^{2},$$ (4)

where ∂ is the roughness matrix.

To minimize the objective function, the partial derivative of the inversion model parameter ***m*** in Eq (3) is calculated, with ∇ *Um* = 0 The iterative formula corresponding to the inversion model parameter is as follows:

$$[{\left(WJ\right)}^{T}WJ+\mu \partial^{T}\partial ]\Delta m_{k}={\left(WJ\right)}^{T}W\Delta d_{k},$$ (5)

Where Δ*m*_*k*_ is the modification of the *k*-th inversion model variable, Δ*d*_*k*_ is the residual vector between the *k*-th model response and the measured data, and *J* is the Jacobian matrix.

The inversion equation can be iterated according to Eq (5) until the optimal solution is reached, after which the electrical parameters of the underground medium are obtained.

#### 2.2.2. Inversion of multiple electrical layer models

The two- and three-layer models were used to verify the rationality of the algorithm, whereas the four-layer model was used to illustrate the fitting situation of the inversion process. The parameters are listed in Table 1.

**Table 1:** Parameters of the inversion models.

Two-layer model	D-type	Layer 1: *ρ*1 = 100 Ω·m, *h*1 = 50 m Layer 2: *ρ*2 = 20 Ω·m
Three-layer model	K-type	Layer 1: *ρ*1 = 100 Ω·m, *h*1 = 100 m Layer 2: *ρ*2 = 400 Ω·m, *h*2 = 100 m Layer 3: *ρ*3 = 100 Ω·m
	H-type	Layer 1: *ρ*1 = 100 Ω·m, *h*1 = 100 m Layer 2: *ρ*2 = 20 Ω·m, *h*2 = 100 m Layer 3: *ρ*3 = 100 Ω·m
Four-layer model	HK-type	Layer 1: *ρ*1 = 120 Ω·m, *h*1 = 200 m Layer 2: *ρ*2 = 20 Ω·m, *h*2 = 400 m Layer 3: *ρ*3 = 200 Ω·m, *h*3 = 300 m Layer 4: *ρ*4 = 120 Ω·m
	KH-type	Layer 1: *ρ*1 = 20 Ω·m, *h*1 = 300 m Layer 2: *ρ*2 = 120 Ω·m, *h*2 = 300 m Layer 3: *ρ*3 = 20 Ω·m, *h*3 = 200 m Layer 4: *ρ*4 = 200 Ω·m

**Fig 3:**
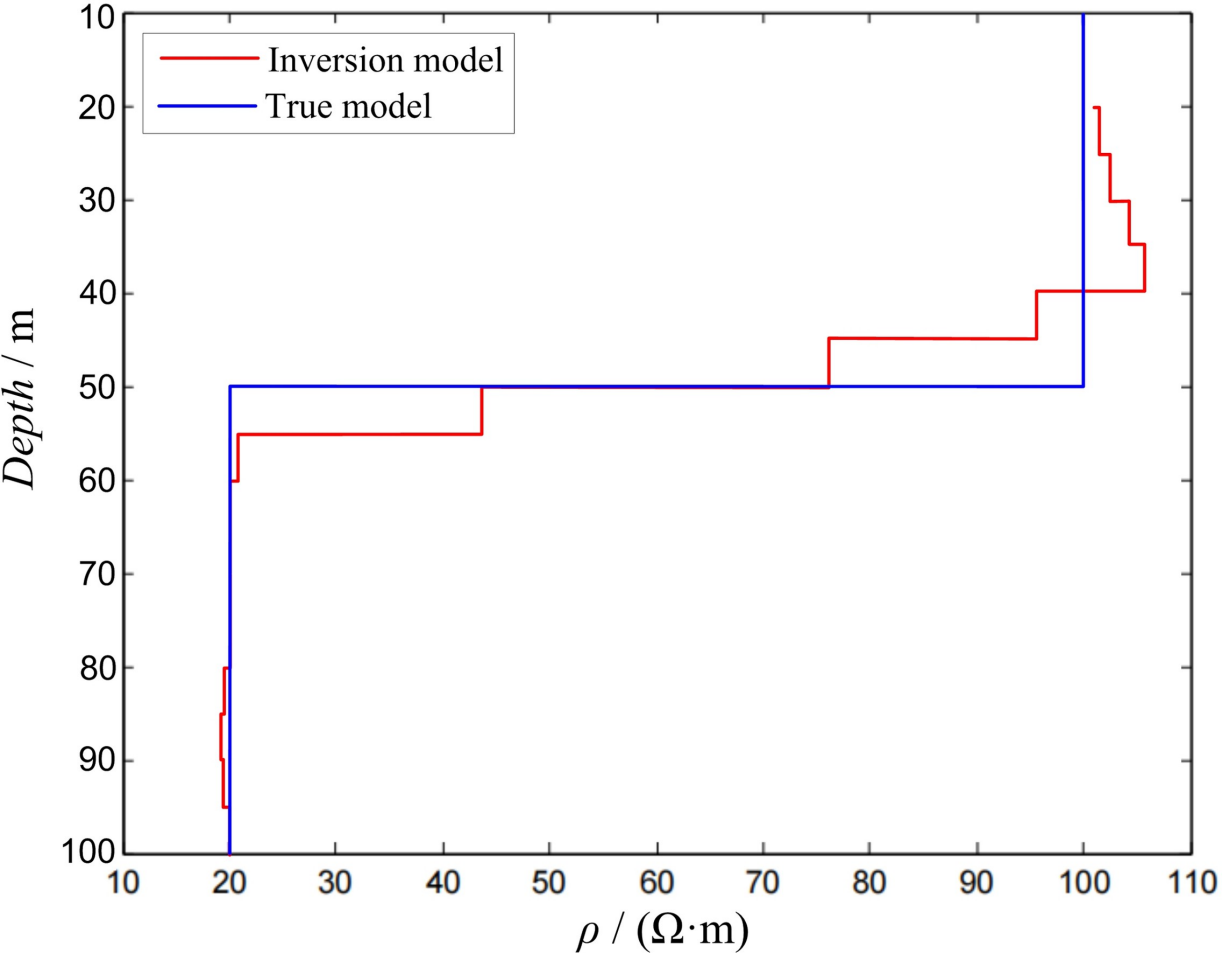
Inversion of two-layer D-type model.
- Three-layer model
  In the three-layer K-type geoelectric model, the side length of the transmitting loop was 300 × 300 m, ρ1 = 100 Ω·m, ρ2 = 400 Ω·m, ρ3 = 100 Ω·m, h1 = 100 m, and h2 = 100 m. The response value was obtained using forward calculation and substituted into the inversion program for calculation. During the inversion process, the layer thickness of the inversion model was 25 m, and there were 13 layers (25–350 m) in total. All the layers had a fixed initial resistivity of 200 Ω·m. The inversion calculation results are shown in Fig 4.

- Two-layer model
  In the two-layer D-type geoelectric model, the side length of the transmitting loop was 100 × 100 m, ρ1 = 100 Ω·m, ρ2 = 20 Ω·m, and h1 = 50 m. The response value was obtained using forward calculation and substituted into the inversion program for calculation. During the inversion process, the layer thickness of the inversion model was 5 m, and there were 15 layers (20–95 m) in total. All the layers had a fixed initial resistivity of 80 Ω·m. The inversion calculation results are shown in Fig 3.

**Fig 4:**
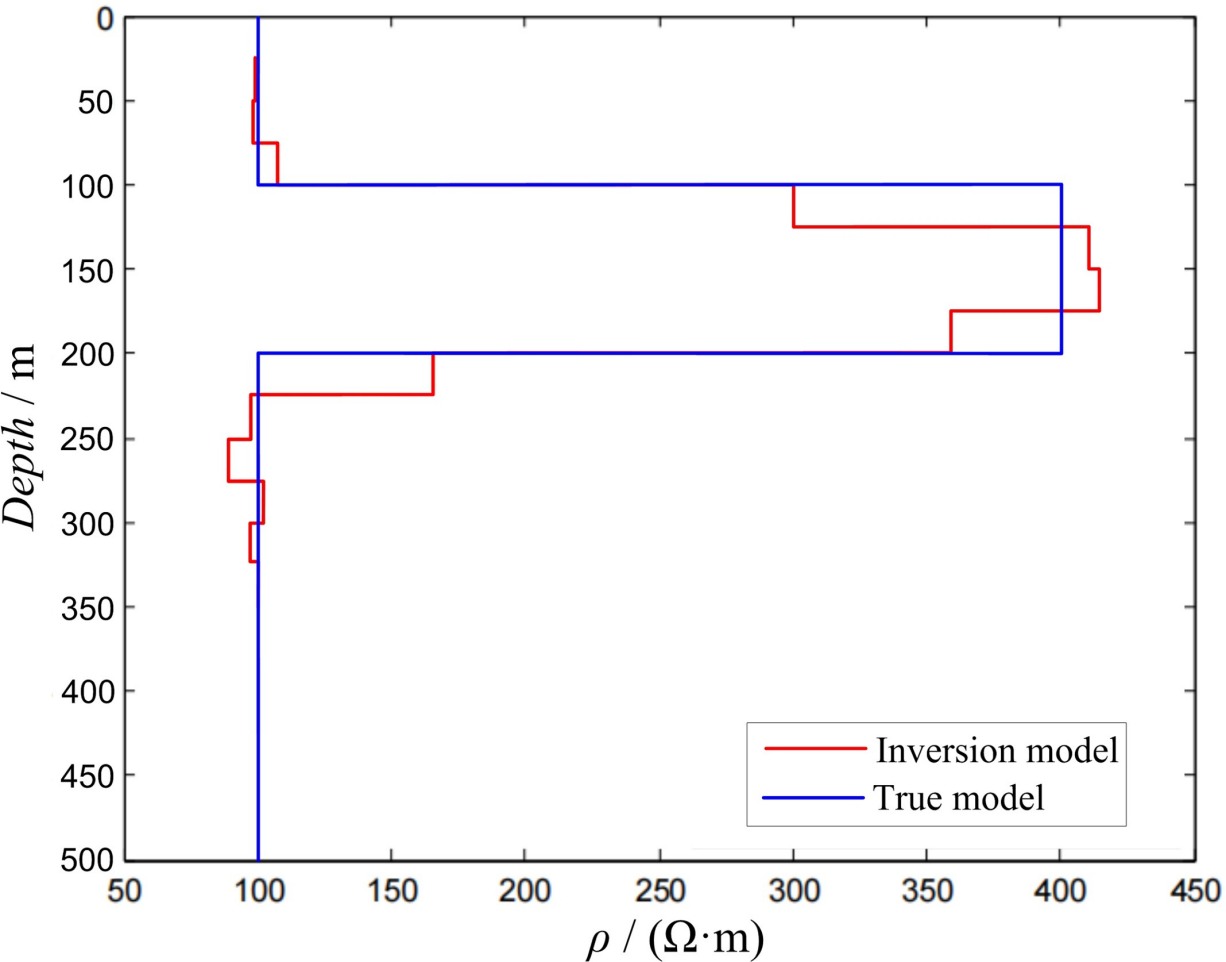
Inversion of three-layer K-type model.
  In the three-layer H-type geoelectric model, the side length of the transmitting loop was 100 × 100 m, ρ1 = 100 Ω·m, ρ2 = 20 Ω·m, ρ3 = 100 Ω·m, h1 = 100 m, and h2 = 100 m. The response value was obtained using forward calculation and substituted into the inversion program for calculation. During the inversion process, the layer thickness of the inversion model was 25 m, and there were 13 layers (25–350 m) in total. All the layers had a fixed initial resistivity of 200 Ω·m. The inversion calculation results are shown in Fig 5.

**Fig 5:**
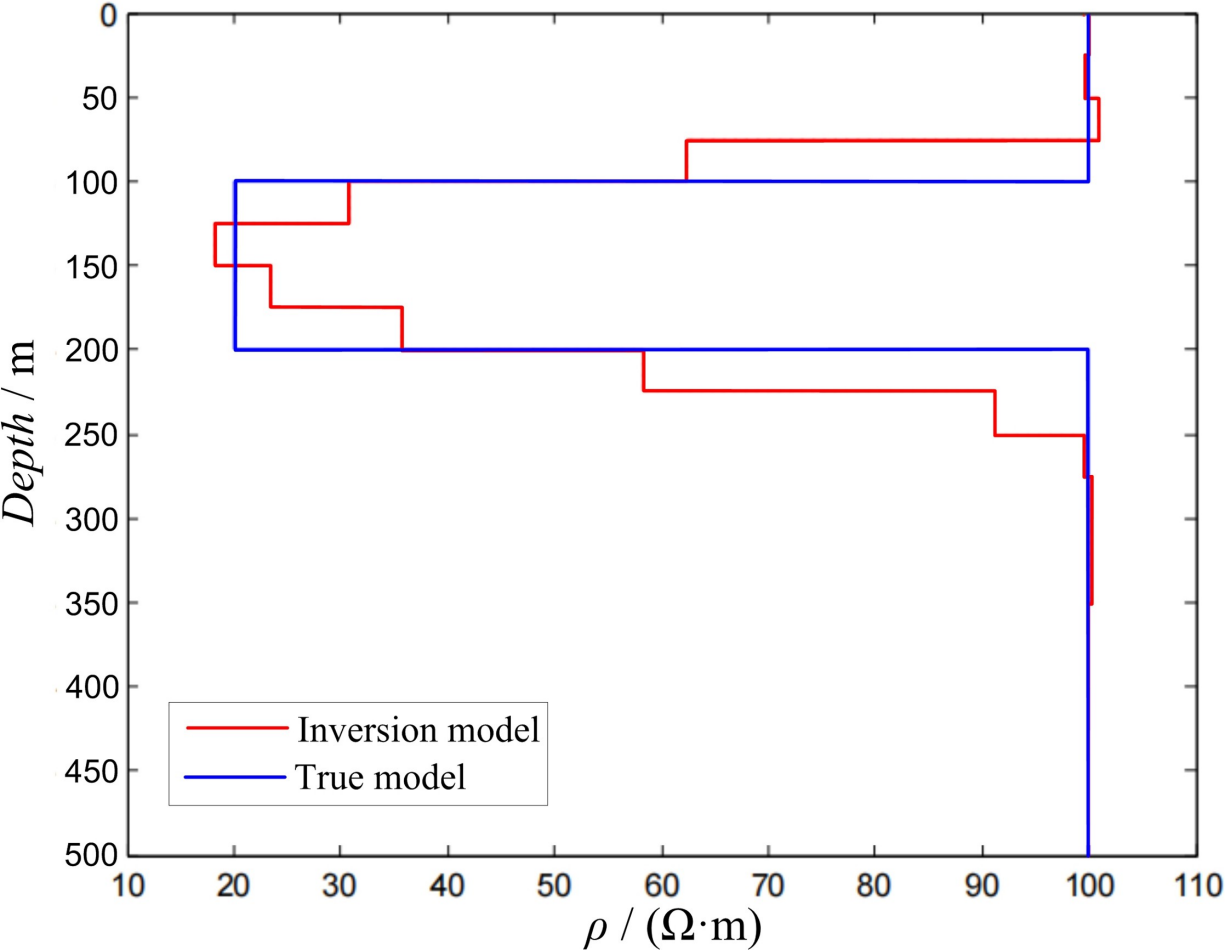
Inversion of three-layer H-type model.
- Four-layer model
  Using the four-layer geodetic model as an example, the multiple iterative inversion process of the 1D Occam inversion algorithm was analyzed.
  In the four-layer HK-type geoelectric model, the side length of the transmitting loop was 600 × 600 m, ρ1 = 120 Ω·m, ρ2 = 20 Ω·m, ρ3 = 200 Ω·m, ρ4 = 120 Ω·m, h1 = 200 m, h2 = 400 m, and h3 = 300 m. The response value was obtained using forward calculation and substituted into the inversion program for calculation. During the inversion process, the layer thickness of the inversion model was 25 m, and there were 38 layers (50–1,000 m) in total. All the layers had a fixed initial resistivity of 100 Ω·m, and the respective numbers of inversion iterations were 1, 3, 5, 7, 9, and 10. The inversion calculation results are shown in Fig 6, where it can be observed that as the number of iterations increases, the inversion model continuously fits the forward model. When the number of iterations is 9 or 10, the inversion results are similar, clearly reflecting the existence of different electrical layers. The inversion resistivity of the low-resistivity layer can be made consistent using the forward model; however, the inversion resistivity of the high-resistivity layer is different from that of the forward model. In the forward model, the resistivity of the high-resistivity layer is 200 Ω·m, whereas after 10 iterations, the inversion resistivity is 180 Ω·m, which is different from the real resistivity by 10%.

**Fig 6:**
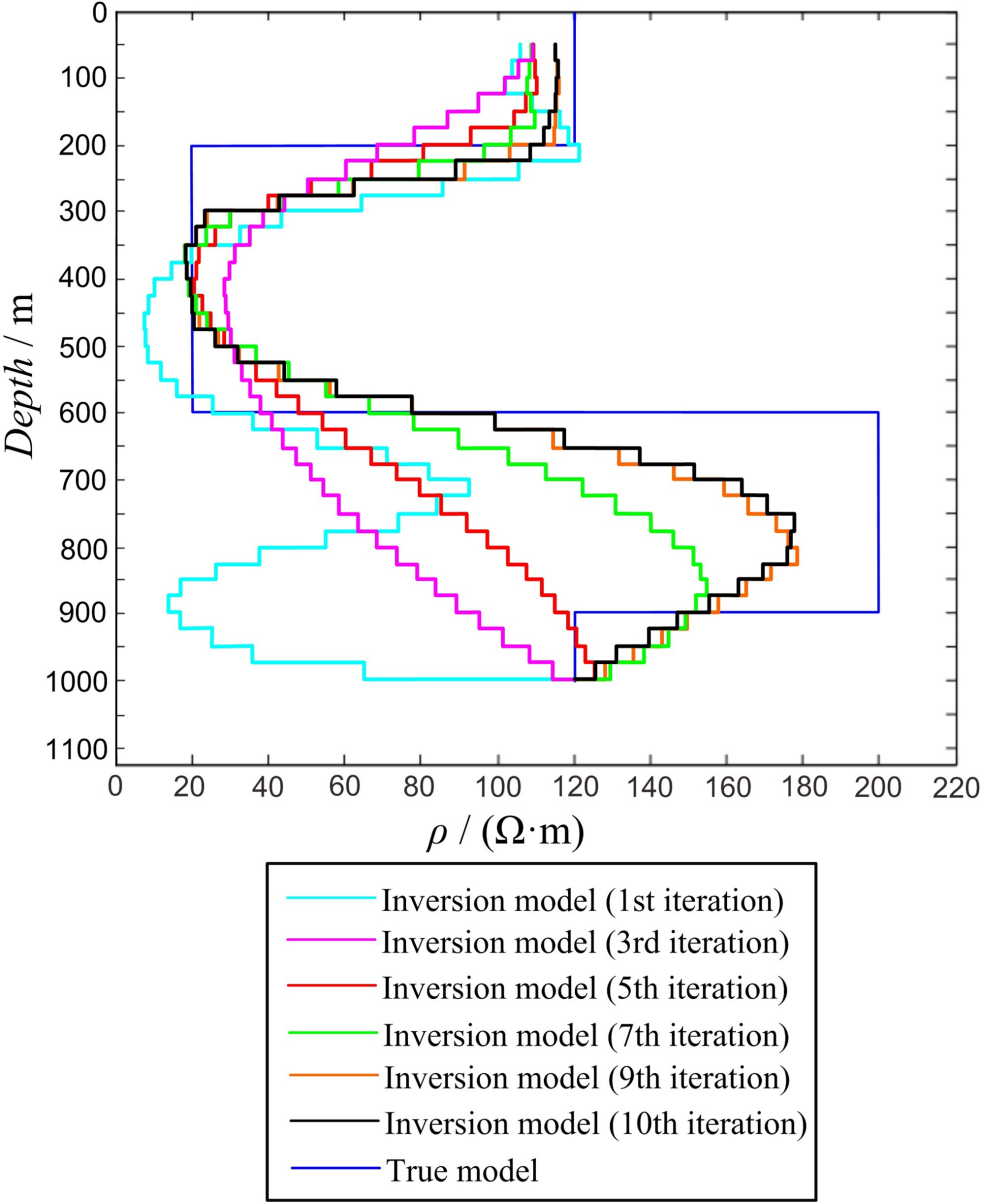
Inversion of four-layer HK-type model.
  In the four-layer KH-type geoelectric model, the side length of the transmitting loop was 600 × 600 m, ρ1 = 20 Ω·m, ρ2 = 180 Ω·m, ρ3 = 20 Ω·m, ρ4 = 200 Ω·m, h1 = 300 m, h2 = 300 m, and h3 = 200 m. The response value was obtained using forward calculation and substituted into the inversion program for calculation. During the inversion process, the layer thickness of the inversion model was 25 m, and there were 38 layers (50–1,000 m) in total. All the layers had a fixed initial resistivity of 100 Ω·m, and the respective numbers of inversion iterations were 1, 3, 5, 7, 9, and 10. The inversion calculation results are shown in Fig 7, where it can be observed that as the number of iterations increases, the inversion model continuously fits the forward model. When the number of iterations is 9 or 10, the inversion results are better and similar, which clearly reflects the existence of different electrical layers. Both the inversion resistivities of the high- and low-resistivity layers are consistent with the forward model.

**Fig 7:**
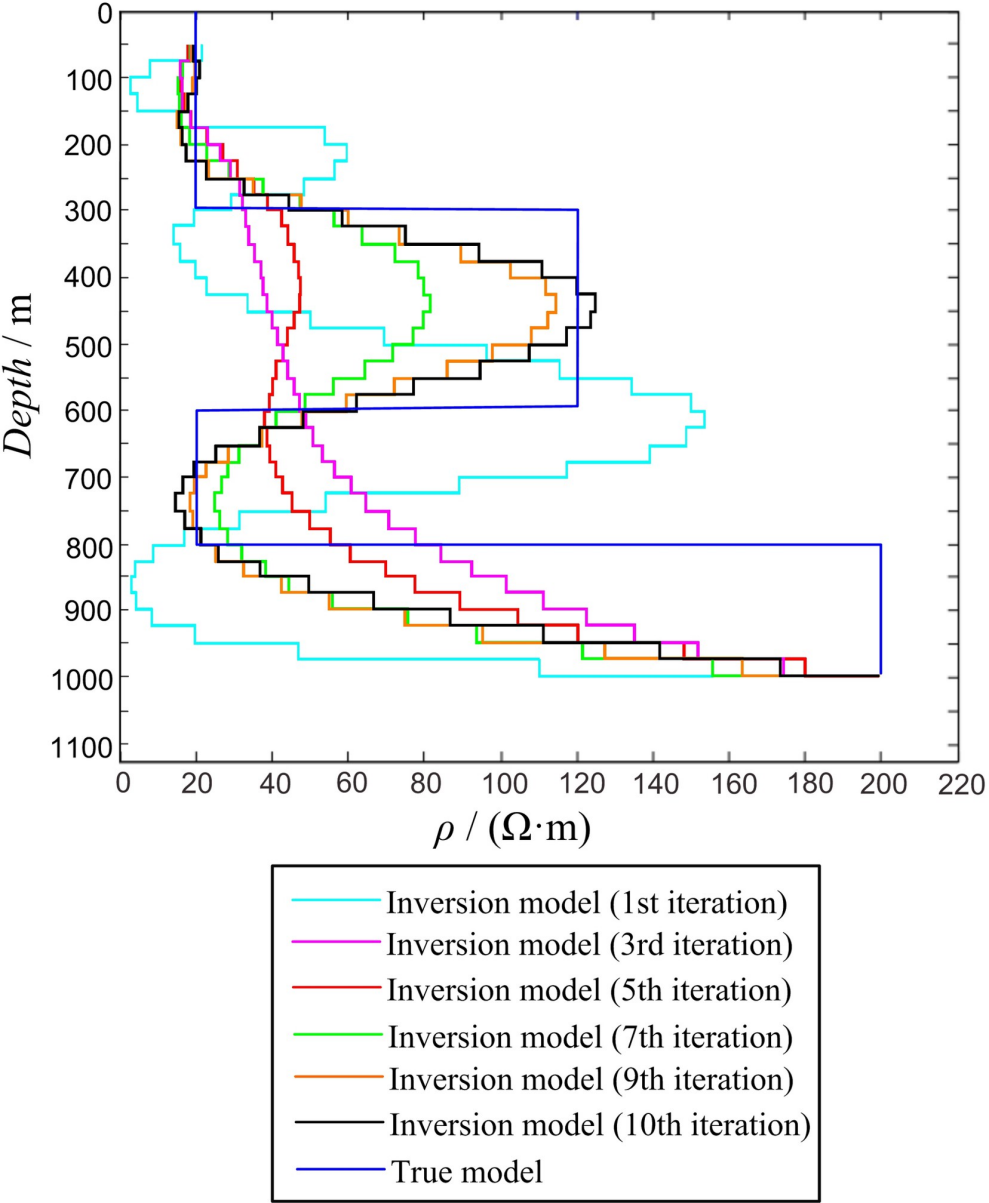
Inversion of four-layer KH-type model.

In summary, in the two- and three-layer models, the 1D structures were clearly resolved using the 1D Occam inversion method, whereas in the four-layer model, the inversion accuracy differed slightly from the real resistivity. When the number of iterations is ≤7, the four-layer structure cannot be effectively displayed after inversion; however, when the number of iterations is >7, the change in the trend of the subsurface structure can be clearly reflected. Overall, the inversion data obtained using the 1D Occam inversion method are satisfactory.

## 3. Experimental analysis

In this study, to verify the effectiveness of the 1D Occam inversion method in detecting multiple electrical layers in overlying coal seams, we conducted a transient electromagnetic exploration project in the Datong Coalfield, Shanxi Province, China.

### 3.1. Geological setting

**Fig 8:**
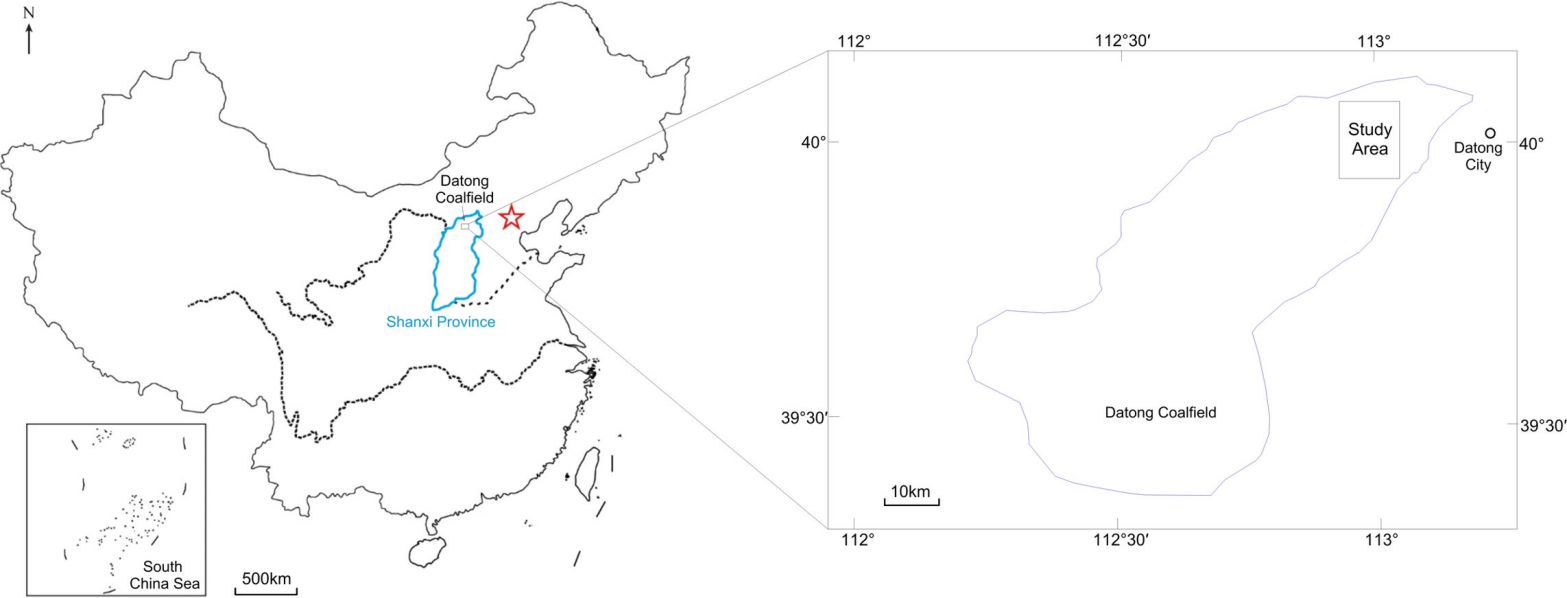
Location of the study area (cited from Zhu et al., [24]).

The study area was located on the northeastern edge of the Datong Coalfield, Shanxi Province, China (Fig 8). The surface of the study area was a river alluvial plain with flat terrain. The strata from shallow to deep are Quaternary, Neogene, Permian, and Carboniferous, and the main coal-bearing strata are the Carboniferous Shanxi Formation. The coal seam in this area was buried at a depth of approximately 750 m. In the strata, high- and low-resistivity layers are interlaced, and there are several different electrical properties overlying the coal seam.

The corresponding multilayer geoelectric model was established by collecting borehole data in the study area. From the characteristics of the borehole data, it can be seen that the Quaternary stratum is approximately 250 m thick, primarily comprising sandy clay, silty sand, and sand layers, and its resistivity is lower than that of the lower Neogene stratum. There are gravel layers containing limestone in the upper part of the Neogene stratum, and some thin layers of coarse sandstone are present in the lower parts of the stratum, which are reflected by the high resistivity as a whole. The total thickness of the Neogene stratum is approximately 200 m. Below the Neogene stratum, the Permian stratum has a lower resistivity than the Neogene stratum. The lowest part is the Carboniferous coal-bearing stratum, which has higher resistivity than the Permian stratum.

In summary, the electrical characteristics of the formation in the study area can be summarized as “low-high-low-high resistivity” from shallow to deep, signifying a multi electrical-layer-staggered formation (Table 2).

**Table 2:** Generalized stratigraphy of the study area.

System	Formation	Thickness (m)	Rock description	Resistivity Characteristic
Quaternary		0–250	Alluvial layers composed	Low
			of sandy clay and silty	
Neogene	Jingle Formation	0–120	Gravel and limestone	High
	Hannuoba	0–80	Coarse sandstone	
	Formation			
Permian	Shiqianfeng Formation	0–60	Sandstone and Siltstone	Low
	Upper Shihezi Formation	0–90	Sandstone and sandy mudstone	
	Lower Shihezi Formation	0–70	Sandstone, siltstone, and mudstone	
Carboniferous	Shanxi Formation	0–110	Sandstone, sandy mudstone, mudstone, and coal; the main coal seam is contained in this formation	High
	Taiyuan Formation	0–50	Sandstone, mudstone, and limestone at the bottom	

### 3.2. Data acquisition device and parameters

For data collection, the GDP-32^II^ Multifunction Workstation by Zonge Co. was used, and the transmitting source was a large fixed-source loop device. The transmitting parameters were as follows: the transmitting loop size, current, and frequency were 840 × 840 m, 10 A, and 32 Hz, respectively. The receiving parameters were as follows: the receiving probe matched with GDP-32^II^ was used to collect data from the center of the transmitting loop with a receiving area of 280 × 280 m and a data sampling delay time of 320 μs. The survey line and point distances were 80 m and 40 m, respectively.

### 3.3. Analysis of inversion effect

#### 3.3.1. Data inversion for a single point next to the borehole

**Fig 9:**
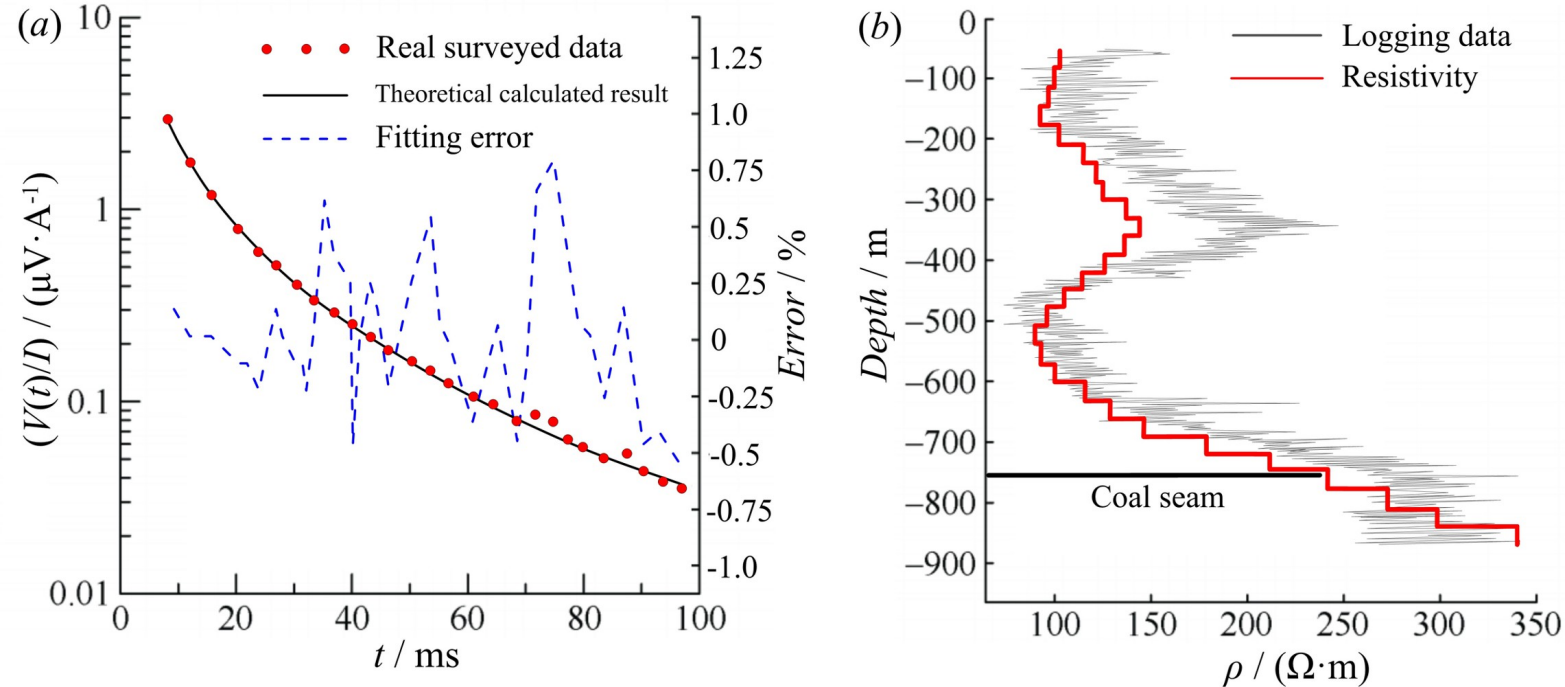
Inversion results of logging data next to the borehole. (*a*) Survey data and fitting curve and (*b*) inverted resistivity curve and borehole logging curve.

As mentioned above, borehole data were collected, and an inversion calculation was performed, the results of which was compared with the borehole resistivity logging curve. During the inversion process, the layer thickness of the inversion model was set to 30 m, with 27 layers (50–860 m). As shown in Fig 9A and Table 3, the minimum, maximum, and average fitting errors were 0.018%, 0.826%, and 0.398%, respectively. Fig 9B displays the inversion results, which generally show the electrical characteristics of “low-high-low-high resistivity” from the shallow to deep strata. Compared with the borehole logging curve (Fig 9B), the inversion results are consistent with the characteristics of the logging curve, based on which the inversion effect can be considered to be good.

**Table 3:** Statistics of synthetic and real data.

Layer number	Thickness (m)	Depth (m)	Real surveyed data (μV·A^−1^)	Theoretical calculated result (μV·A^−1^)	Fitting error (%)
1	30	50–80	3.217	3.025	0.192
2	30	80–110	1.837	1.819	0.018
3	30	110–140	1.243	1.218	0.025
4	30	140–170	0.795	0.927	−0.132
5	30	170–200	0.587	0.811	−0.224
6	30	200–230	0.557	0.542	0.098
7	30	230–260	0.406	0.512	−0.103
8	30	260–290	0.356	0.483	−0.141
9	30	290–320	0.291	0.457	−0.246
10	30	320–350	0.467	0.431	0.657
11	30	350–380	0.424	0.407	0.353
12	30	380–410	0.389	0.385	0.384
13	30	410–440	0.265	0.363	−0.433
14	30	440–470	0.348	0.343	0.266
15	30	470–500	0.321	0.324	−0.193
16	30	500–530	0.316	0.290	0.325
17	30	530–560	0.096	0.156	−0.158
18	30	560–590	0.080	0.122	−0.446
19	30	590–620	0.091	0.089	0.665
20	30	620–650	0.080	0.065	0.826
21	30	650–680	0.066	0.063	0.114
22	30	680–710	0.060	0.057	0.121
23	30	710–740	0.050	0.053	−0.253
24	30	740–770	0.054	0.049	0.187
25	30	770–800	0.043	0.044	−0.422
26	30	800–830	0.039	0.039	−0.468
27	30	830–860	0.032	0.034	−0.523

#### 3.3.2. Data inversion for a whole line

**Fig 10:**
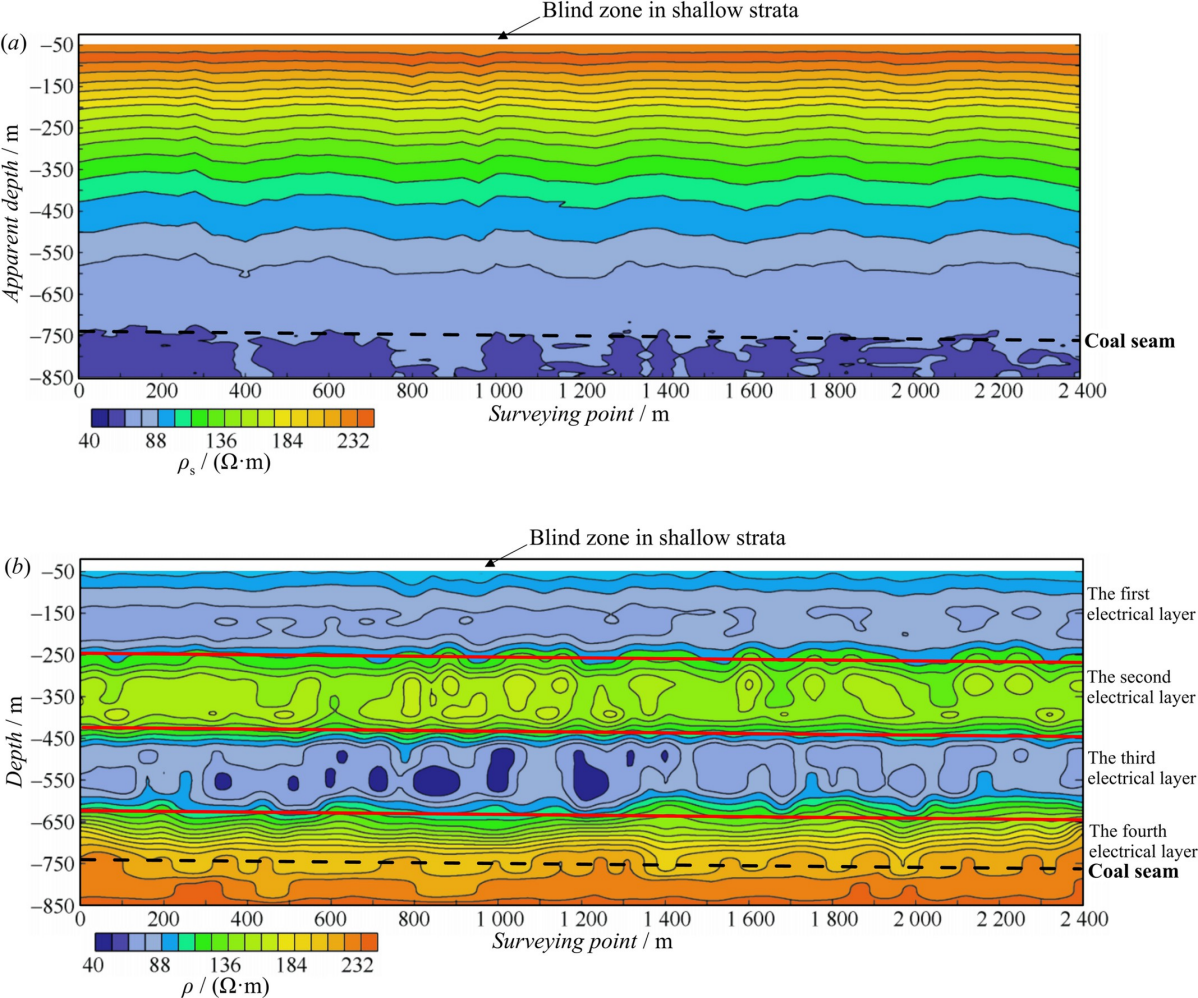
Comparison of profiles before and after inversion. (*a*) The “apparent resistivity–apparent depth” profile before inversion and (*b*) the “resistivity–depth” profile after inversion.

For the data of the entire survey line, the apparent resistivity profile was drawn, as shown in Fig 10A. In this figure, the apparent resistivity contours are distributed in layers, and the quality of the data, which lays the foundation for the inversion calculation, is good. In the vertical direction, the electrical trend of “high-low resistivity” is shown from shallow to deep layers, and the resistivity value is the lowest at the bottom of the profile (at a depth of 850 m), which is inconsistent with the geophysical characteristics of the study area; hence, the electrical layer could not be distinguished.

According to the 1D Occam inversion method, the data in Fig 10A were inverted, and the inversion result was shown in Fig 10B. As shown in Fig 10B, the overall characteristics of the profile were significantly different from those in Fig 10A, and the inversion depth reached 850 m. In the inversion profile, the shallow data (at a depth of 50–250 m) showed evident low resistivity, which was inferred to be the first electrical layer. As the depth increased, a high-resistivity layer appeared at a depth of 250–420 m, which was inferred to be the second electrical layer. This was followed by a stronger low-resistivity layer with a thickness of approximately 200 m from a depth of 420 m, which was inferred to be the third electrical layer. The bottom of the profile is a high-resistivity layer (at a depth 620–−850 m), which is inferred to be the fourth electrical layer. The overall electrical characteristics of the profiles were consistent with their geophysical characteristics.

By comparing Fig 10A and 10B, it can be seen that the inversion results are in close agreement with the actual formation resistivity, indicating that this inversion method has a strong ability to identify multiple electrical layers. In addition, the inversion results have a high resolution, which can lay a strong foundation for subsequent interpretation.

## 4. Conclusion

When using the late apparent resistivity and apparent depth formulas, the TEM data cannot truly reflect the geoelectric information in the detection of multiple large-depth electric layers. To solve this problem, a 1D Occam inversion method is required. Using two-, three-, and four-layer theoretical geoelectric models, it can be seen that the inversion method can distinguish between high and low resistivities. Using the 1D Occam method to invert the TEM data, satisfactory results were achieved, which were verified using the sample project in the Datong Coalfield, Shanxi Province, China.

## Supporting information

S1 Data (RAR)
